# ChatGPT vs Gemini: Comparative Accuracy and Efficiency in CAD-RADS Score Assignment from Radiology Reports

**DOI:** 10.1007/s10278-024-01328-y

**Published:** 2024-11-11

**Authors:** Matthew Silbergleit, Adrienn Tóth, Jordan H. Chamberlin, Mohamed Hamouda, Dhiraj Baruah, Sydney Derrick, U. Joseph Schoepf, Jeremy R. Burt, Ismail M. Kabakus

**Affiliations:** 1 Division of Cardiothoracic Imaging, Department of Radiology and Radiological Science, Clinical Science Building, Medical University of South Carolina, 96 Jonathan Lucas Street, Suite 210, MSC 323, Charleston, SC 29425 USA; 2 Division of Cardiothoracic Imaging, Department of Radiology and Radiological Science, University of Utah School of Medicine, Salt Lake City, UT USA

**Keywords:** LLM, CAD-RADS, AI, Coronary CTA, ChatGPT, Gemini

## Abstract

This study aimed to evaluate the accuracy and efficiency of ChatGPT-3.5, ChatGPT-4o, Google Gemini, and Google Gemini Advanced in generating CAD-RADS scores based on radiology reports. This retrospective study analyzed 100 consecutive coronary computed tomography angiography reports performed between March 15, 2024, and April 1, 2024, at a single tertiary center. Each report containing a radiologist-assigned CAD-RADS score was processed using four large language models (LLMs) without fine-tuning. The findings section of each report was input into the LLMs, and the models were tasked with generating CAD-RADS scores. The accuracy of LLM-generated scores was compared to the radiologist’s score. Additionally, the time taken by each model to complete the task was recorded. Statistical analyses included Mann–Whitney *U* test and interobserver agreement using unweighted Cohen’s Kappa and Krippendorff’s Alpha. ChatGPT-4o demonstrated the highest accuracy, correctly assigning CAD-RADS scores in 87% of cases (*κ* = 0.838, *α* = 0.886), followed by Gemini Advanced with 82.6% accuracy (*κ* = 0.784, *α* = 0.897). ChatGPT-3.5, although the fastest (median time = 5 s), was the least accurate (50.5% accuracy, *κ* = 0.401, *α* = 0.787). Gemini exhibited a higher failure rate (12%) compared to the other models, with Gemini Advanced slightly improving upon its predecessor. ChatGPT-4o outperformed other LLMs in both accuracy and agreement with radiologist-assigned CAD-RADS scores, though ChatGPT-3.5 was significantly faster. Despite their potential, current publicly available LLMs require further refinement before being deployed for clinical decision-making in CAD-RADS scoring.

**Supplementary Information:**

The online version contains supplementary material available at 10.1007/s10278-024-01328-y.

## Introduction

Through the implementation of pre-trained language models, the efficacy of natural language processing has rapidly advanced [1]. This has opened numerous possibilities regarding their potential clinical, educational, or research applications within healthcare [2, 3]. The theory behind pre-trained language models is twofold. First, there is initial training via large volume, unsupervised learning. This is followed by an optional, fine-tuning stage where the pre-trained model is adjusted to perform a specific task [4]. Fine-tuned large language models (LLMs) have been developed for many medical subspecialties, from mammography [4] to traditional Chinese medicine [5].

While fine-tuned LLMs have demonstrated promising performance for interpretation and augmentation of radiology reports, the cost and resource intensity of the fine-tuning process remains a barrier [6]. Generic LLMs are publicly available and do not require additional cost or expertise to train and utilize, making them a more feasible option for most practices and healthcare systems presently [7]. With multiple publicly available LLMs, it can be difficult to determine which model to use. While there have been comparisons between several of the publicly available LLMs for other radiology applications, such as BI-RADS (Breast Imaging Reporting and Data System) and LI-RADS (Liver Imaging Reporting and Data System) category assignments [8, 9], there has yet to be a comprehensive analysis of the accuracy of the most prevalent LLMs in generating CAD-RADS (Coronary Artery Disease Reporting and Data System) scores and recommendations based on data from radiology reports.

The CAD-RADS score provides a systematic framework for reporting coronary computed tomography angiography (CCTA) findings, aiding in clinical decision-making and patient management [10]. Accurate extraction of CAD-RADS scores from radiology reports by LLMs could streamline workflow and enhance clinical efficiency. However, the challenges associated with training models for specific tasks, such as the need for large, annotated datasets and extensive computational resources, highlight the potential advantages of leveraging generic LLMs for this purpose.

We aimed to evaluate the performance of ChatGPT-3.5, ChatGPT-4o, Google Gemini, and Google Gemini Advanced in generating CAD-RADS scores based on accompanying radiology reports. Additionally, we compared response times of these LLMs. The findings from this study will provide valuable insights into the feasibility of using LLMs for CAD-RADS extraction and their potential role in enhancing radiology reporting practices. With further refinement, LLMs could assist radiologists by automating portions of the report generation process, specifically the impression section, which could improve reporting efficiency and consistency. However, significant steps are needed before these tools are ready for routine clinical use. A critical aspect of their deployment would involve U.S. Food and Drug Administration approval, ensuring patient data privacy by transmitting only anonymized data, free of protected health information, to the LLMs. In this framework, the LLMs would provide a preliminary Reporting and Data System score and impression, which radiologists could then review and adjust as needed. This dual approach (combining automated assistance with radiologist oversight) has the potential to enhance clinical workflow while maintaining high standards of patient care and data security.

## Materials and Methods

### *Design and Compliance*

This study was designed as a single-institute, retrospective analysis aimed at evaluating the performance of LLMs in generating CAD-RADS scores from heart CT angiography (CTA) reports. The study was conducted in accordance with institutional guidelines, and Institutional Review Board (IRB) approval was obtained (Pro00136914). Informed consent from the patients was waived due to the retrospective nature of the study.

### Clinical Examinations

This study analyzed radiology reports for all CCTA scans performed at a single, tertiary referral center over a 2-week period. All source reports were initially generated by fellowship-trained cardiothoracic radiologists. Each report included a CAD-RADS score assigned by the reporting radiologist. All studies were performed between 15 March 2024 and 1 April 2024. Beginning 1 April, the 100 most recently conducted CTA heart scans were included in this study. A total of 8 reports of congenital heart CTAs were excluded from the study, as these cases do not typically involve detailed coronary artery assessments and CAD-RADS scoring. Statistical analysis demonstrated that a sample size of 64 cases would provide adequate statistical power (80%) for the comparisons we are making. With our current sample of 100 cases, the study achieves a power of approximately 94%.

### Clinical Data and LLM Results

Findings section of each report, excluding protected health information (PHI) and the impression (which contained the CAD-RADS score), was input into the LLMs for analysis. Study date, demographics (gender, age, BMI, and ethnicity), presence of stent or coronary artery bypass grafting (CABG), Fractional Flow Reserve CT (FFR/CT) analysis, and reader-assigned CAD-RADS score were recorded but not shared with LLMs.

We utilized templated coronary computed tomography angiography reports (Online Resource) that were structured to assess each coronary artery individually. The degree of stenosis for each artery was categorized as follows: no stenosis (CAD-RADS 0), minimal stenosis (1), mild stenosis (2), moderate stenosis (3), severe stenosis (4), and occluded (5). The CAD-RADS score for each report was assigned based on the highest degree of stenosis present in any of the coronary arteries.

These initial reports were generated by five fellowship-trained cardiothoracic radiologists, with a final review and confirmation by the senior author. Since this study relied on structured template report based score assignment rather than direct image analysis, it minimized subjectivity inherent in image interpretation. The structured nature of the reports enabled a standardized approach to CAD-RADS scoring based solely on documented findings, thereby enhancing the objectivity of the scoring process.

**Fig. 1:**
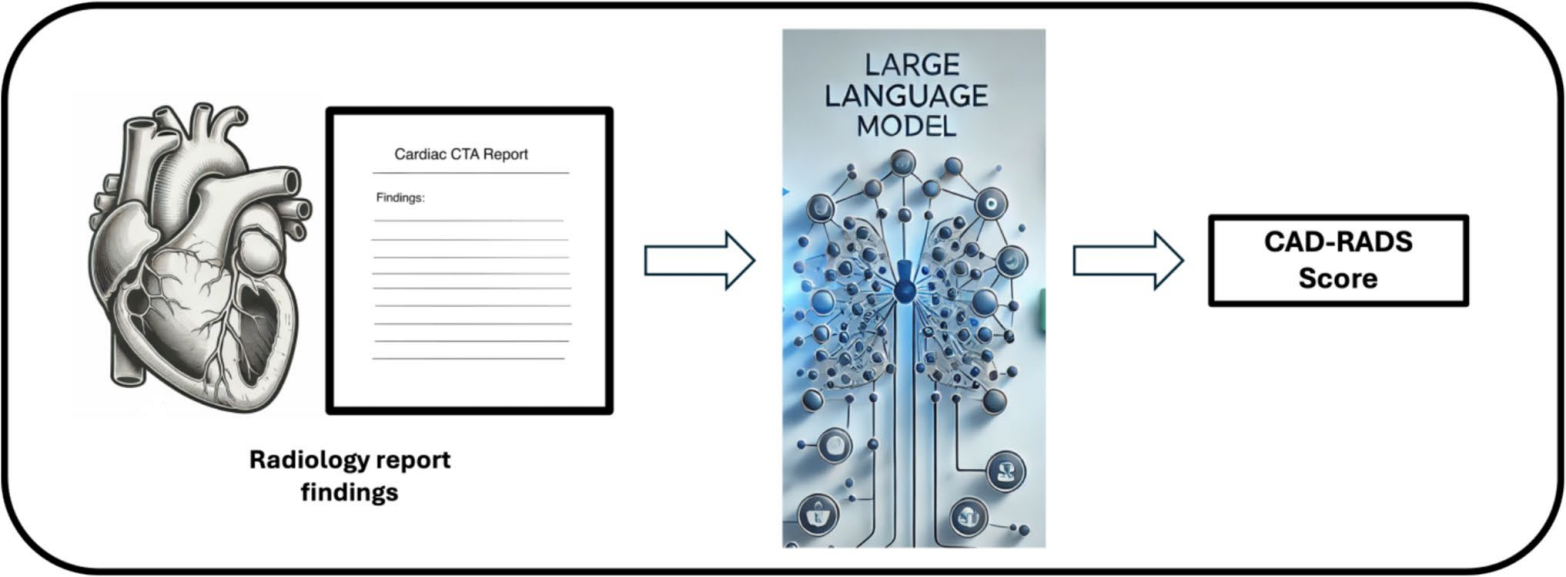
Workflow for CAD-RADS score generation using large language models

Four large language models were evaluated in this study: ChatGPT-3.5, ChatGPT-4o, Google Gemini, and Google Gemini Advanced. These models were not fine-tuned for this specific task. Instead, for each case, a new chat session was initiated, and the findings section of the heart CTA report was shared with the LLM. The LLMs were then asked to assign a CAD-RADS score based on the provided information (What is the CAD-RADS score for the following cardiac CTA report?). All potential patient identifying information was excluded from text entered into LLMs. All findings text input was exactly as it was dictated. LLMs did not have access to any other section of the report or the reader-assigned CAD-RADS score. LLMs did not receive any feedback following their assignment of CAD-RADS score. A new session was started for each report. All the responses, including those that the LLMs failed to assign a score, were saved (Fig. 1). All LLMs were tested for each case at the same time, before moving on to the next case, ensuring similar conditions and internet speeds.

The ground truth for this study was the CAD-RADS score as documented in the original radiology report by board-certified radiologists and confirmed by the senior author. The performance of each LLM was assessed based on its accuracy in generating CAD-RADS scores that matched the ground truth.

In addition to accuracy, the time taken by each LLM to generate a CAD-RADS score was recorded. The time was measured from the moment the findings section was input into the LLM to the receipt of the completed answer. This time measurement allowed for a comparison of the efficiency of each LLM. The time was measured manually using a stopwatch.

### Statistical Analysis

All LLM reports were analyzed for both time and accuracy as compared to the dictating radiologist. Statistical comparisons of time were performed using the Mann–Whitney *U* test for non-parametric samples. Interobserver agreement was calculated using both unweighted Cohen’s Kappa and the Krippendorff Alpha using ordinal weights. Cohen’s kappa was utilized to assess agreement while accounting for chance but not accounting for magnitude of error. Krippendorff’s Alpha accounts for magnitude of error by punishing observations which deviate from the ground truth more severely. Both values are provided (with and without magnitude of error adjustment) as both the absolute agreement and deviation from the ground truth are important values. All analysis was performed in *R* version 4.1.2 (R Foundation for Statistical Programming, Vienna, Austria). In this study, a *p*-value of less than 0.05 was considered statistically significant.

## Results

One hundred sequential patients were included in this study, of which 21 were classified by the dictating radiologist as CAD-RADS 0, 26 as CAD-RADS 1, 21 as CAD-RADS 2, 16 as CAD-RADS 3, 14 as CAD-RADS 4, and 2 as CAD-RADS 5. Patient demographics are summarized in Table 1.

**Table 1:** Demographics and ground truth imaging variables

*N* = 100	Median/IQR | *N*/%
Age	62.5 (20)
Sex	
Female	50 (50)
Male	50 (50)
BMI	28.1 (7.9)
Race	
Asian	2 (2)
Black	27 (27)
Hispanic	1 (1)
White	62 (62)
Other	5 (5)
Unknown	3 (3)
Stent	6 (6%)
CABG	4 (4%)
FFR/CT	2 (2%)
CAD-RADS	
0	21 (21%)
1	26 (26%)
2	21 (21%)
3	16 (16%)
4	14 (14%)
5	2 (2%)

**Fig. 2:**
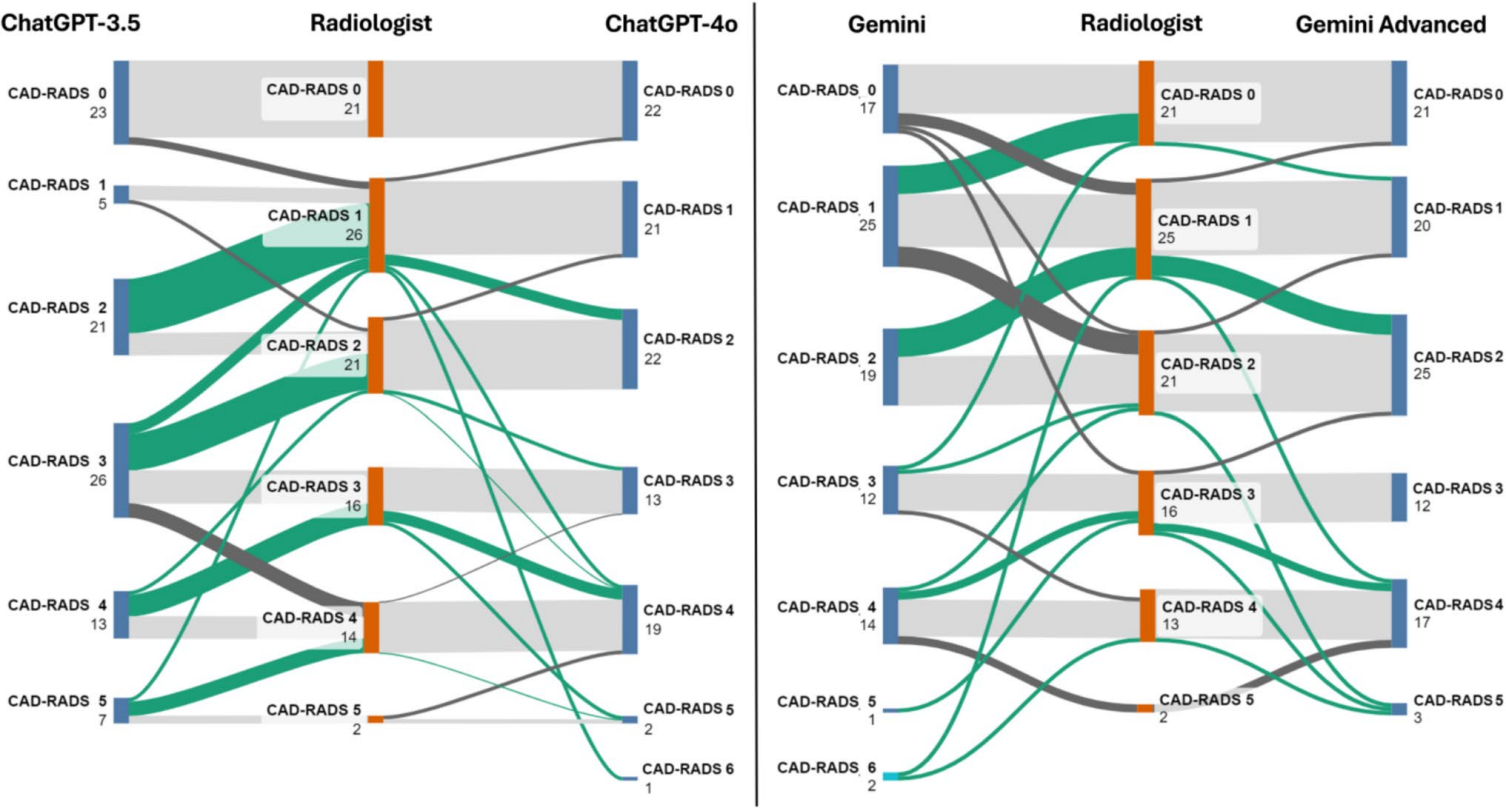
Sankey graph illustrates the comparison between CAD-RADS scores generated by large language models and those assigned by radiologists. The green flows represent instances where the GPT models overestimated the severity of coronary artery disease, leading to higher CAD-RADS scores compared to the radiologists’ assessments. The light gray flows indicate cases where the CAD-RADS scores from the GPT models aligned with those given by radiologists, showing perfect correlation. In contrast, the dark gray flows show instances where the GPT models underestimated the severity of the disease

There was significant variation between the LLMs for qualitative score assessment. ChatGPT-4 was able to assign a score in 99% of cases, compared to ChatGPT-3.5 at 95%, Gemini at 88%, and Gemini Advanced at 97%. ChatGPT-3.5 assigned only 5 (actual *n* = 26) patients to be CAD-RADS 1 while comparatively over assessing (26 vs 16) CAD-RADS 3 and CAD-RADS 5 (7 vs 2). ChatGPT-4o improved on the deficits of ChatGPT-3.5 but still underestimated the number of CAD-RADS 1 scores (21 vs 26) and instead overestimated CAD-RADS 4 (19 vs 14). ChatGPT-4o correctly assessed 2 patients to have a score of CAD-RADS 5. Gemini assigned less cases to all categories except for CAD-RADS 4 (14 vs 14) due 12% failure no-result rate. Gemini Advanced improved upon Gemini’s results with mild underestimation of CAD-RADS 1 and CAD-RADS 3 categories (19 vs 26 and 12 vs 16, respectively) as well as mild overestimation of CAD-RADS 2 and CAD-RADS 4 categories (25 vs 21 and 17 vs 14, respectively) (Table 2, Fig. 2).

**Table 2:** Distribution of CAD-RADS interpretations by radiologists and LLMs

	**CAD-RADS 0**	**CAD-RADS 1**	**CAD-RADS 2**	**CAD-RADS 3**	**CAD-RADS 4**	**CAD-RADS 5**
Radiologist	21	26	21	16	14	2
ChatGPT-3.5	23	5	21	26	13	7
ChatGPT-4o*	22	21	22	13	19	2
Gemini**	17	25	19	12	14	1
Gemini Advanced	21	19	25	12	17	3

^*^Assigned one case as “CAD-RADS 6”

^**^Assigned two cases as “CAD-RADS 6”

Out of 100 reports, 6 included information regarding stents, 4 regarding CABG, and 2 regarding FFR/CT analysis (Table 1). ChatGPT-4o and Gemini Advanced added the modifier “S” for stents in 2 out of the 6 patients. None of the LLMs added any additional modifiers for CABG or FFR/CT. Interestingly, Gemini Advanced added the modifier “P” for calcification amount in only 1 case.

**Fig. 3:**
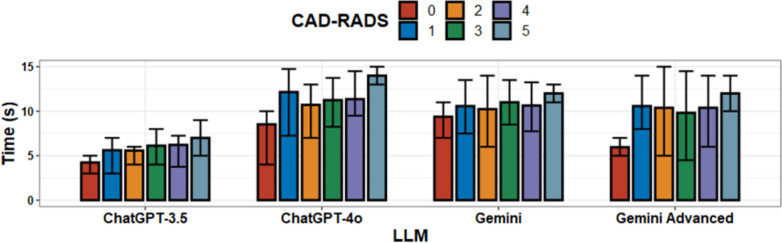
Time to complete task stratified by CAD-RADS score. Generally, time to complete task increases with higher CAD-RADS score

**Fig. 4:**
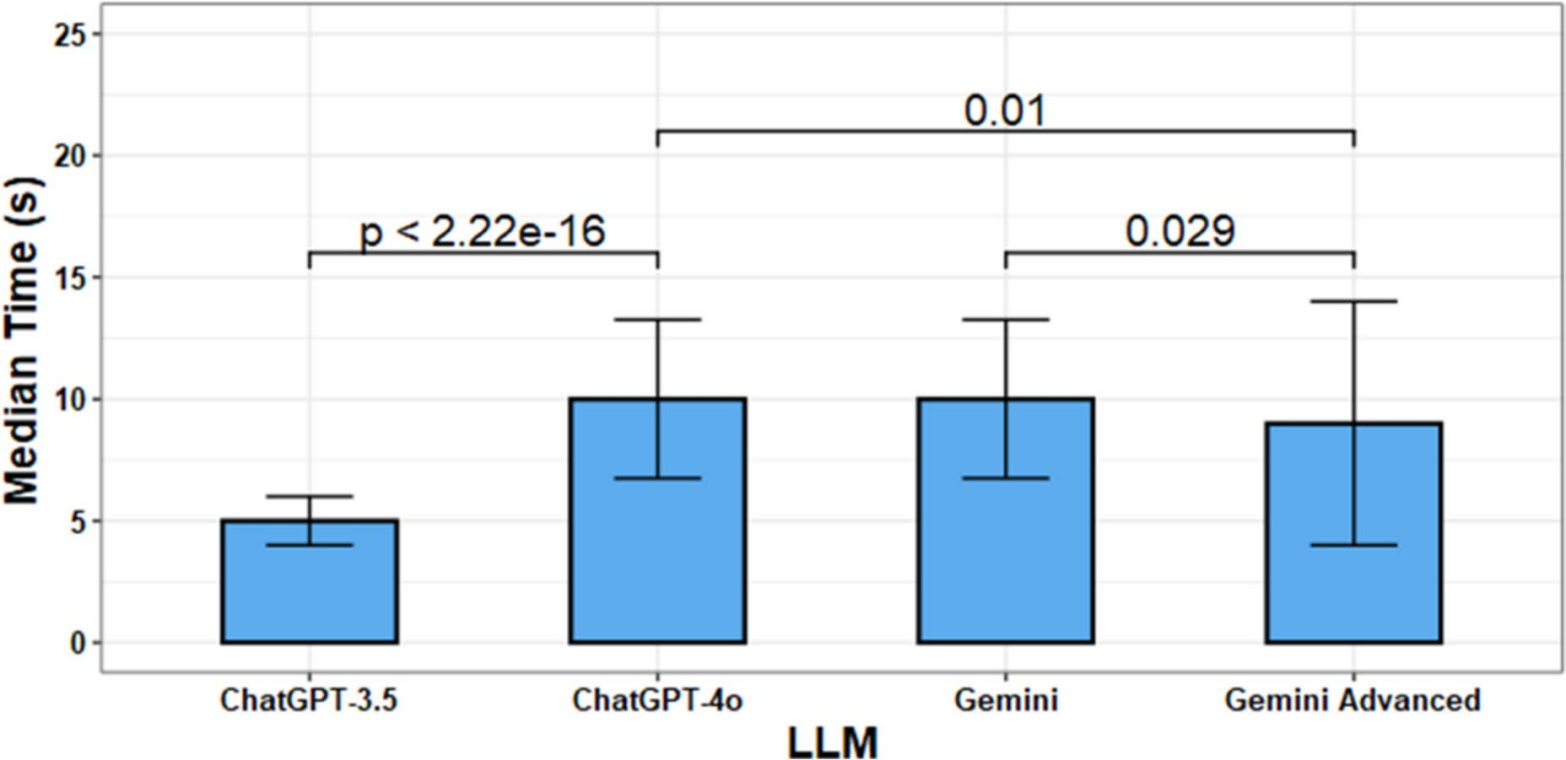
Comparison of time to complete task by LLMs. ChatGPT-3.5 is the fastest LLM (Median Time = 5 s) and is significantly faster than ChatGPT-4o (*p* < 0.001). Gemini Advanced is slightly faster than Gemini (8 s vs 10 s, *p* = 0.029) and ChatGPT-4o (8 s vs 10 s,* p* = 0.01); however, all times are extremely close and likely any difference between LLMs other than ChatGPT-3.5 is imperceptible

All LLMs were quick in completing the assigned tasks. In general, higher categories of CAD-RADS were associated with increased time to completion, but the differences were minimal (Median Time for CAD-RADS 1 = 8 s, Median Time for CAD-RADS 5 = 9.5 s) and probably imperceptible, likely due to small sample size (Fig. 3). ChatGPT-3.5 was the fastest of all the models tested with median time to complete task of 5 s. This was significantly faster than that of ChatGPT-4o (10 s, *p* < 0.001). Gemini and Gemini Advanced had similar median times with a slight edge to Gemini Advanced (8 s vs 10 s, *p* = 0.029), but this result is likely imperceptible. Similarly, ChatGPT-4o was slower than Gemini Advanced (10 s vs 8 s, *p* = 0.01) (Table 3, Fig. 4).

**Table 3:** Distribution of time to complete task by LLMs and CAD-RADS score. Time given as Median/IQR, s

	**CAD-RADS 0**	**CAD-RADS 1**	**CAD-RADS 2**	**CAD-RADS 3**	**CAD-RADS 4**	**CAD-RADS 5**
ChatGPT-3.5	4 (1) s	5 (2) s	5 (1) s	6 (2) s	5.5 (1.8) s	7 (2) s
ChatGPT-4o	7 (3) s	11 (3.8) s	10 (3) s	11 (2.8) s	12 (2.5) s	14 (1) s
Gemini	9 (2) s	10.5 (3) s	10 (4) s	11 (2.5) s	10.5 (2.8) s	12 (1) s
Gemini Advanced	6 (1) s	11 (3.8) s	10 (5) s	9.5 (5) s	10 (4) s	12 (2) s

**Fig. 5:**
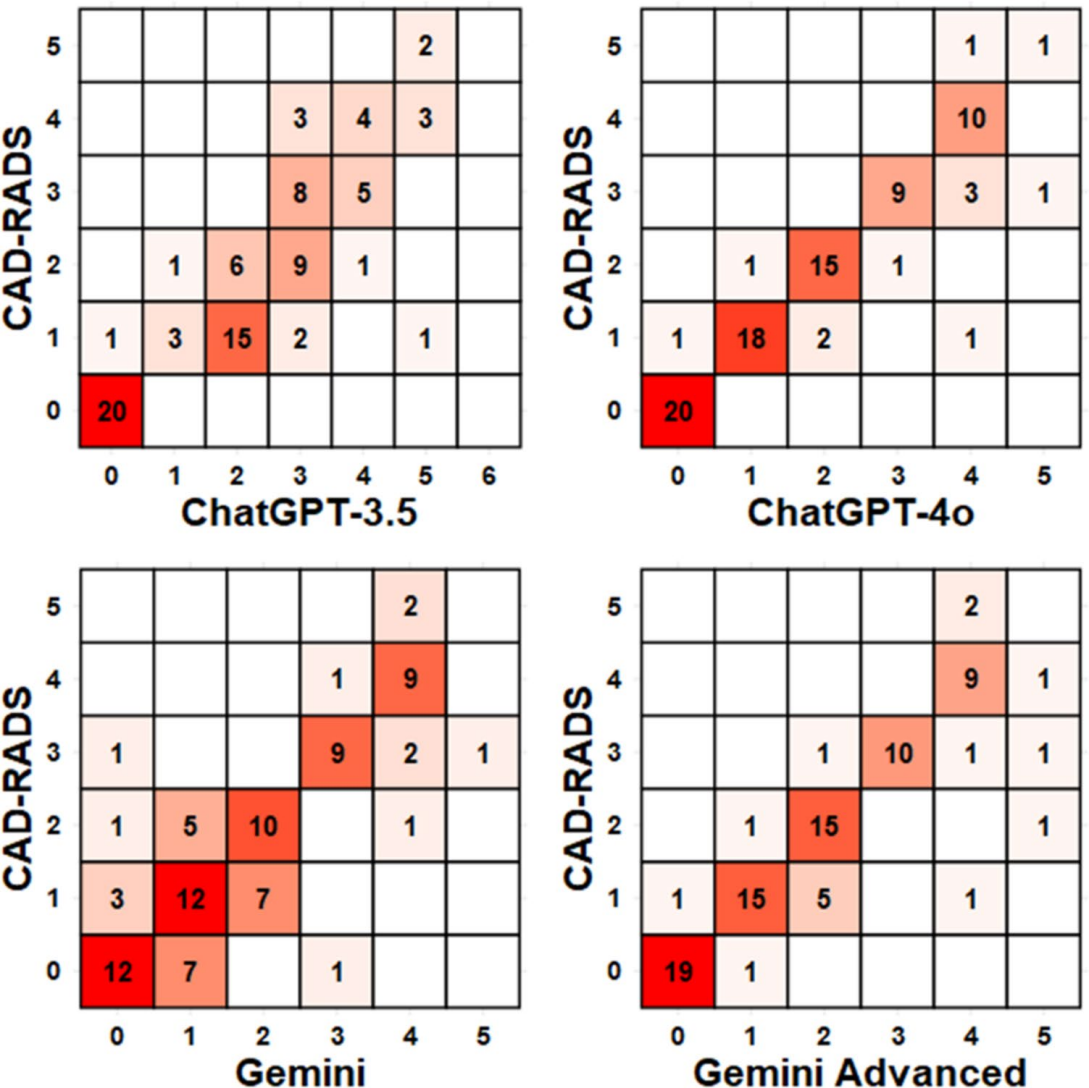
Visual representation of agreement statistics. ChatGPT-4o and Gemini Advanced performed the best with most observations either correct or within one category of the ground truth. More errors are observed for the lower CAD-RADS assessments

Raw accuracy and interobserver agreement were assessed for each LLM with the radiology report as the ground truth. Both ChatGPT-4o and Gemini Advanced outperformed their previous generation counterparts in all metrics (ChatGPT, 87.0% vs 50.5%; Gemini, 82.6% vs 61.1%). Both ChatGPT-4o and Gemini Advanced approached nearly perfect alpha values (ChatGPT-4o *α* = 0.886, 95% CI 0.781–0.991; Gemini Advanced *α* = 0.897, 95% CI 0.832–0.962). While these values are similar, ChatGPT-4o 1 observation with no results while Gemini Advanced had three, although ChatGPT-4o classified 1 result as “CAD-RADS 6,” a rating which does not exist. Regarding unweighted agreements, ChatGPT-4o held a slight advantage (0.838, 95% CI 0.757–0.920 vs 0.784, 95% CI = 0.691–0.877), suggesting slightly more robust results to chance (Table 4). Visualizations of the agreements are given as the confusion matrices in Fig. 5.

**Table 4:** Unbalanced accuracy, unweighted Cohen’s *κ*, and ordinal-weighted Krippendorff’s *α* for comparison of accuracy and agreement of CAD-RADS score

	Accuracy	Unweighted *κ*	Krippendorff *α*	No Result
ChatGPT-3.5	0.505 (0.401–0.610)	0.401 (0.285–0.517)	0.787 (0.701–0.873)	5%
ChatGPT-4o	0.870 (0.788–0.923)	0.838 (0.757–0.920)	0.886 (0.781–0.991)	1%
Gemini	0.611 (0.503–0.712)	0.513 (0.385–0.641)	0.737 (0.618–0.870)	12%
Gemini Advanced	0.826 (0.740–0.896)	0.784 (0.691–0.877)	0.897 (0.832–0.962)	3%

## Discussion

While tailored LLMs have been demonstrated to have utility for specific tasks within radiology [6], there is limited data regarding the applicability of the more widely available, generic LLM chatbots. The goal of this study was to assess the accuracy of four of the most popular, publicly available LLMs (ChatGPT-3.5, Chat GPT-4o, Gemini, and Gemini Advanced) in assigning a CAD-RADS score based solely on the text of the accompanying radiology report. In a sample of 100 reports, ChatGPT-4o was the most accurate when compared to the gold standard of a fellowship-trained cardiothoracic radiologist, while ChatGPT-3.5 was the fastest but least accurate. ChatGPT and Gemini generally tended to overestimate the severity of CAD-RADS scores compared to radiologists, assigning patients to higher categories. This trend was notably pronounced with ChatGPT 3.5 and Gemini. However, an improvement was observed with ChatGPT-4o and Gemini Advanced, which showed a more accurate alignment with radiologists’ assessments (Fig. 2). Gemini failed to assign a score to 10% of the cases, while Gemini Advanced had a failure rate of 2% and ChatGPT-3.5 at 5%. ChatGPT-4o, however, successfully assigned a score to 99% of the cases. This finding also demonstrates the superiority of the ChatGPT algorithm over Gemini.

While the LLMs demonstrated reasonable accuracy in assigning primary CAD-RADS categories, they did not perform as well in detecting and adding modifiers, such as those for stents, CABG, or FFR/CT analysis. This limitation is likely due to the models’ general training data, which may lack the specialized focus needed to accurately recognize and apply these specific radiology modifiers consistently.

ChatGPT-3.5 had the fastest response time, while ChatGPT-4o exhibited the slowest. This was an interesting finding, considering ChatGPT-4o is a more advanced model. The slower response time is likely due to ChatGPT-4o providing more detailed answers compared to ChatGPT-3.5. There was no significant difference between the response times of Gemini and Gemini Advanced, although Gemini Advanced offered more detailed responses.

While the highest performing LLM (ChatGPT-4o) in this study showed moderate agreement with the true CAD-RADS score, they fell well behind human–human interobserver agreement [11, 12]. Interobserver agreement for CAD-RADS has historically been excellent. Maroules et al. demonstrated high levels of consensus among both early career and expert readers, with early career readers achieving an intra-class correlation of 0.904, while expert readers had an even higher degree of agreement with an intra-class correlation of 0.925 [11]. Additionally, Ippolito et al. also demonstrated excellent interobserver agreement among two readers assigning CAD-RADS scores [12]. Importantly, in both of these studies, all readers had access to the source images and generated independent reports prior to reaching a final score. This study differs in that the LLMs did not have access to any images, only a select section of the radiology report. Given the strict lexicon utilized in CAD-RADS, it is possible that human–human interobserver agreement would be even higher if CAD-RADS scoring was based solely on the radiologic report and images were excluded.

These results fit with the trend of publicly available chatbots underperforming humans in radiologic tasks regarding complex decision making. Both ChatGPT and Gemini have demonstrated limitations related to management and clinical decision making, including liver imaging recommendations based on clinical and laboratory data [13]. Fervers et al. reported low accuracy for ChatGPT-3.5 in determining LI-RADS scores from German written liver MRI imaging reports, correctly classifying at most 53% of the lesions [9]. ChatGPT and Gemini (then Bard) also underperformed traditional search engines Google and Bing, regarding questions related to lung cancer imaging [14]. A recent study by Cozzi et al. evaluated the performance of LLMs in assigning BI-RADS categories based on breast imaging reports written in three different languages. They found that while there was moderate agreement between LLM-assigned and human-assigned BI-RADS scores, there were also significant discrepancies that would negatively impact clinical management.

One of the primary reasons the LLMs failed to achieve human-level accuracy in CAD-RADS assessment likely stems from their inability to fully interpret the nuanced context within radiology reports. Although CAD-RADS scoring is text-based, the complexity of individual cases introduces subtle distinctions that require clinical expertise, which the LLMs lack. Furthermore, while trained on a broad spectrum of general language data, these models may struggle with specialized medical terminology and specific radiology lexicon critical for consistent scoring. Another challenge lies in the inherent variability in report language, even within structured reports, which can affect the models’ capacity to apply CAD-RADS criteria as precisely as human radiologists.

Our additional analysis revealed that certain factors frequently contribute to incorrect CAD-RADS scores by LLMs. For instance, reports with longer, more complex sentences and detailed descriptors (such as mentions of bypass grafts, stents, plaque vulnerability, plaque type, or segment length) often led to misinterpretation. The presence of multiple modifiers and extensive descriptions can complicate sentence structure, overwhelming the model’s parsing capabilities. Additionally, nuanced terms not directly tied to CAD-RADS categories may divert the model’s focus, resulting in incorrect score assignments. These observations suggest that LLMs may particularly struggle with complex language and cases involving multiple interventions, impacting their scoring accuracy.

A crucial component of this study was the utilization of publicly available LLMs, in contrast to fine-tuned language models. Pre-trained and fine-tuned LLMs have demonstrated utility for narrow, specific tasks [15]. However, this comes at a cost. Pre-trained and fine-tuned LLMs require markedly more time and resources than publicly available chatbots such as ChatGPT and Gemini. Fine-tuned LLMs in particular require very specific data and tend to have a narrow scope while being focused on one specific task. Additionally, there are vastly different regulatory requirements for medical grade LLMs. As medical LLMs are regulated by the FDA, there are a number of checks and balances relating to both the initial implementation and continued updating of LLMs that must be considered. This greatly raises the barrier for production of approved LLMs, especially as each fine-tuned LLM has a narrow clinical scope. These barriers may prevent smaller radiology groups from utilizing LLMs, potentially excluding them from a tool that has the potential to improve practice efficiency. This emphasizes the appeal of chatbots such as ChatGPT and Gemini. If these publicly available, free, easy to use, and easy to access LLMs showed acceptable accuracy in performing specific radiologic tasks, they would have the potential to dramatically change practice.

It must be noted that LLMs operate on external servers, which may lead to vulnerabilities in data security and privacy breaches if patient information is inadvertently shared. Although we utilized de-identified data in this study, any clinical application would require robust mechanisms to ensure that protected health information remains secure. Additionally, the integration of these models into clinical workflows would necessitate compliance with stringent regulations, such as HIPAA in the USA, to safeguard sensitive information. These factors highlight the need for careful consideration of privacy and security protocols before publicly available LLMs can be widely implemented in healthcare settings.

There were a few limitations to this study. First, the LLMs only had access to a single, specific section of the radiology report, lacking further information from the images, clinical data, or the entire report of the patient’s scan being evaluated. The LLMs were reliant on the reading radiologist to use appropriate terminology to accurately describe the images with vocabulary they could utilize to assign a CAD-RADS score, even though the reading radiologist did have access to the complete set of diagnostic information. Second, each report was initially read by only one radiologist. While all readers involved were fellowship-trained in cardiothoracic radiology and CAD-RADS scoring has been shown to have excellent interrater reliability, this study did lack the internal control of multiple readers verifying each report. Third, all studies were obtained from a single institution over a relatively short period of time. This limited the geographic and temporal scope of the patient population, which may affect the generalizability of the findings. The sample size was relatively small, which may affect the generalizability of the results, particularly for higher CAD-RADS score categories. Our study does not involve direct clinical implementation; however, our findings provide valuable insights into the models’ accuracy and efficiency, which are critical precursors to safe and effective clinical integration. The evolving nature of open-access language models influences the applicability of our findings over time. While our results are specific to the versions tested, they establish a valuable baseline for understanding the capabilities and limitations of publicly accessible LLMs in CAD-RADS scoring.

In conclusion, ChatGPT-4o outperformed ChatGPT-3.5, Google Gemini, and Google Gemini Advanced in terms of accuracy and agreement with a fellowship-trained cardiothoracic radiologist when assigning CAD-RADS scores based on radiology reports. While less accurate, ChatGPT-3.5 was the fastest of the four LLMs evaluated. Although ChatGPT-4o in particular shows promising results, this study demonstrates that current publicly available LLMs are not ready to be used in clinical decision making related to CAD-RADS scoring.

## Supplementary Information

Below is the link to the electronic supplementary material.

Supplementary file1 (DOCX 17 KB)

## Data Availability

Data generated or analyzed during the study are available from the corresponding author by request.
