# Supplementary material for: ChatGPT vs Gemini: Comparative Accuracy and Efficiency in CAD-RADS Score Assignment from Radiology Reports
Source: J Imaging Inform Med. 2024 Nov 11;38(4):2303–11. doi: 10.1007/s10278-024-01328-y (PMC12343400; doi:10.1007/s10278-024-01328-y)
Supplement: Supplementary file 1 — Supplementary file1 (DOCX 17 KB) [file 10278_2024_1328_MOESM1_ESM.docx]

**CTA HEART STRUCTURED REPORT**

**FINDINGS:**

**CORONARY CT ANGIOGRAM**

**Left Main Coronary Artery:** No stenosis/Minimal stenosis/Mild stenosis/Moderate stenosis/Severe stenosis/Occluded

**Left Anterior Descending Coronary Artery:** No stenosis/Minimal stenosis/Mild stenosis/Moderate stenosis/Severe stenosis/Occluded

**Left Circumflex Coronary Artery:** No stenosis/Minimal stenosis/Mild stenosis/Moderate stenosis/Severe stenosis/Occluded

**Right Coronary Artery:** No stenosis/Minimal stenosis/Mild stenosis/Moderate stenosis/Severe stenosis/Occluded

This is a right/left-dominant system.

**CARDIAC CHAMBERS AND VALVES**

**Left atrium:** Qualitatively normal left atrial size.

**Left ventricle:** Normal in size. Normal myocardial thickness without areas of hypoattenuation.

**Right atrium:** Qualitatively normal right atrial size.

**Right ventricle**: Normal size and wall thickness.

**Mitral valve:** The mitral valve leaflets are thin and mobile, without stenosis or prolapse.

**Aortic valve:** The aortic valve is tricuspid. The aortic valve leaflets are thin and mobile, without stenosis or prolapse.

**Tricuspid valve:** The tricuspid valve leaflets appear thin and mobile without stenosis or prolapse.

**Pulmonic valve:** Pulmonic valve leaflets are thin and mobile, without evidence of stenosis.

No evidence of intracardiac filling defects. No evidence of atrial or ventricular septal discontinuity.

No evidence of pericardial effusion or thickening.

**VASCULATURE**

**Systemic veins:**

**Pulmonary veins:**

**Pulmonary arteries:** No pulmonary embolism. Normal size main and branch pulmonary arteries without focal stenosis.

**Aorta:** Normal aortic root and ascending aorta. Left arch with normal branching pattern. Descending aorta is normal in course and caliber. No evidence of intramural hematoma, penetrating ulcer, dissection, or aneurysm.

**CHEST**

**Airways/Lungs/Pleura:** Normal airways. No evidence of acute pulmonary or pleural disease.

**Mediastinum:** No lymphadenopathy.

**Soft tissues and bones:** No acute bony abnormality. No suspicious osseous lesions.

Upper abdomen: Unremarkable.

**IMPRESSION:**

No evidence of coronary artery disease.

Normal cardiac chambers and visualized valves. Normal systolic LV function without regional wall motion abnormality.

**CAD-RADS ()**
